# Warfarin versus direct oral anticoagulants for treating left ventricular thrombus: a systematic review and meta-analysis

**DOI:** 10.1186/s12959-021-00259-w

**Published:** 2021-02-01

**Authors:** Tarun Dalia, Shubham Lahan, Sagar Ranka, Amandeep Goyal, Sara Zoubek, Kamal Gupta, Zubair Shah

**Affiliations:** 1 Department of Cardiovascular Medicine, The University of Kansas Medical Center, Kansas City, Kansas, USA; 2 University College of Medical Sciences, New Delhi, India; 3 Department of Pharmacology, The University of Kansas Medical Center, Kansas City, Kansas, USA

**Keywords:** Left ventricular thrombus, Warfarin, Anticoagulation, DOAC/NOAC, Relative risk

## Abstract

**Background:**

Left ventricular thrombus (LVT) is not uncommon and pose a risk of systemic embolism, which can be mitigated by adequate anticoagulation. Direct oral anticoagulants (DOACs) are increasingly being used as alternatives to warfarin for anticoagulation, but their efficacy and safety profile has been debated. We aim to compare the therapeutic efficacy and safety of DOACs versus warfarin for the treatment of LVT.

**Methodology:**

We systematically searched PubMed/Medline, Google Scholar, Cochrane library, and LILCAS databases from inception to 14th August 2020 to identify relevant studies comparing warfarin and DOACs for LVT treatment and used the pooled data extracted from retrieved studies to perform a meta-analysis.

**Results:**

We report pooled data on 1955 patients from 8 studies, with a mean age of 61 years and 59.7 years in warfarin and DOACs group, respectively. The pooled odds ratio for thrombus resolution was 1.11 (95% CI 0.51–2.39) on comparing warfarin to DOAC, but it did not reach a statistical significance (*p* = 0.76). The pooled risk ratio (RR) of stroke or systemic embolization and bleeding in patients treated with warfarin vs DOACs was 1.04 (95% CI 0.64–1.68; *p* = 0.85), and 1.15 (95% CI 0.62–2.13; *p* = 0.57), respectively; with an overall RR of 1.09 (95% CI 0.70–1.70; *p* = 0.48) for mortality.

**Conclusions:**

DOACs appears to be non-inferior or at least as effective as warfarin in the treatment of left ventricular thrombus without any statistical difference in stroke or bleeding complications.

**Supplementary Information:**

The online version contains supplementary material available at 10.1186/s12959-021-00259-w.

## Introduction

Left ventricular thrombus (LVT) can be seen as a complication post myocardial infarction (MI) and also in certain non-ischemic cardiomyopathies [1]. Dating back to pre-perfusion era, the incidence of LVT following an MI used to range from 21% to as high as 46% [2–4]. However, with the advent of re-perfusion techniques, the incidence of LVT has reduced substantially. Depending upon the accuracy of the modality used for diagnosis, current data suggests that the incidence of LVT varies from 4 to 15% [5, 6]. These LVT are particularly notorious for complicating the course of illness because of their propensity of getting dislodged and causing systemic embolization or stroke, which can be decreased with appropriate anticoagulation therapy [7]. Although the current guidelines suggest that the choice of anticoagulation therapy to be used for LVT is a vitamin-K antagonist such as warfarin [8, 9], off-label use of direct oral anticoagulants (DOACs) is becoming popular among both patient and physicians [10, 11]. The effectiveness of DOACs in treating LV thrombus is controversial, with some studies favoring their use and others not [11–14]. The large scale studies and metanalysis comparing the effectiveness and, safety of the DOACs vs warfarin in treatment of LVT are lacking. Thus, we sought to perform this systematic review and meta-analysis to compare the effectiveness and safety of warfarin vs DOACs for the treatment of LVT.

## Methods

This systematic review was conducted in concordance with the preferred reporting items for systematic reviews and meta-analyses (PRISMA) guidelines [15]. (A PRISMA checklist has been included in e-Table 1 in Supplement-1).

**Table 1:** Details of included studies and baseline characteristics of patients in warfarin group and DOACs group

**Authors**	**Year**	**Region**	**Study design**	**Study period**			**Sample size (n)**		**Age (years)**		**Males (%)**	
							Warfarin	DOAC	Warfarin	DOAC	Warfarin	DOAC
Bass et al	2019	United States	Retrospective	Sept 2012 – Oct 2018			769	180	70.9	69.4	63	65.6
Daher et al	2020	France	Retrospective	Jan 2010 – Aug 2019			42	17	63	62.4	61	57
Jaidka et al	2018	Canada	Retrospective	Not specified			37	12	75	75	61.3	57
Robinson et al	2020	United States	Retrospective	Oct 2013 – Mar 2019			236	121	72	77.7	58.2	58
Jones et al	2020	United Kingdom	Observational	Not specified			60	41	85	80.4	60.8	58.7
Yunis et al	2020	United States	Retrospective	Jan 2014 – April 2019			200	64	NR	NR	NR	NR
Ali et al	2020	United States	Retrospective	Jan 2012 – Jan 2019			60	32	58	59	81.6	81.3
Iqbal et al	2020	United Kingdom	Retrospective	Dec 2012 – June 2018			62	22	89	91	62	62
**Hypertension (%)**		**Diabetes (%)**		**Smokers (%)**		**STEMI (%)**		**Assessment of Thrombus resolution**	**Type of DOAC used in the study**	**Follow-up duration**	**Primary end-point**	
Warfarin	DOAC	Warfarin	DOAC	Warfarin	DOAC	Warfarin	DOAC					
NR	NR	NR	NR	NR	NR	57.6	42.8	NR	Not specified	Not specified	Incidence of thromboembolic stroke in patients receiving DOACs compared to warfarin.	
40.5	59	21.4	12	59.5	59	74	88	TTE	Apixaban Rivaroxaban Dabigatran	Not specified	Response to DOACs and VKAs	
48.6	16.7	18.9	8.3	35	50	100	100	TTE	Not specified	Not specified	Treatment and 6-month outcomes (embolic events, bleeding, and thrombus resolution).	
75	71	39	29.8	NR	NR	62.7	54.5	TTE	Apixaban Rivaroxaban Dabigatran	351 days	Risk of stroke or systemic embolization.	
36.4	60.5	16.7	18.4	33.3	21	86.7	87.8	TTE or CMR	Apixaban Rivaroxaban Edoxaban	2.2 year	Left ventricular thrombus resolution.	
NR	NR	NR	NR	NR	NR	NR	NR	Not specified	Not specified	2 years	Survival and freedom from bleeding and from stroke and systemic embolism.	
NR	NR	30	37.5	62	53	NR	NR	TTE	Not specified	1 year	Stroke or systemic embolism and thrombus resolution.	
29	41	31	86	50	45	89	82	CMR (initially) & TTE (follow-up)	Apixaban Rivaroxaban Dabigatran	3 years	Thromboembolic events and bleeding.	

Abbreviations; CMR: Cardiac magnetic resonance; DOAC: Direct oral anticoagulant; NR: Not recorded; TTE: Transthoracic echocardiogram; STEMI: ST-segment elevation myocardial infarction

### Objectives

To compare DOAC vs warfarin treatment in LVT patients by calculating the pooled effect estimates for:

1. Composite risk of stroke or systemic embolization,
2. Composite risk of thrombus resolution,
3. Composite risk of bleeding complications,
4. Composite risk of all-cause mortality

### Search strategy and study selection

**Fig. 1:**
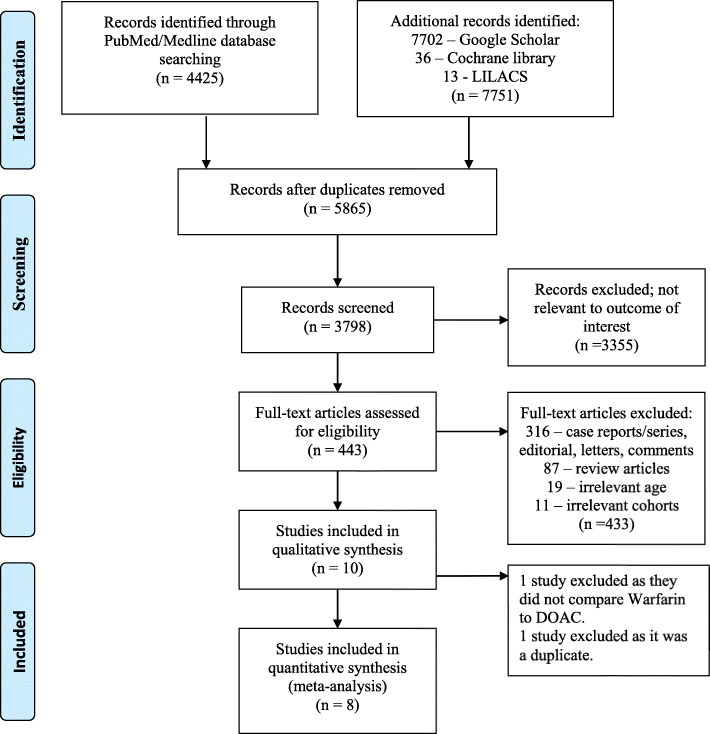
PRISMA flow-diagram illustrating search and inclusion strategy

We systematically searched PubMed/Medline (https://pubmed.ncbi.nlm.nih.gov), Google Scholar, LILACS virtual health library ((https://lilacs.bvsalud.org/en/), and Cochrane library (cochranelibrary.com) databases from inception to 14th August, 2020 to identify and retrieve relevant studies using the following terms: “(left ventricular thrombus) AND (treatment); (left ventricular thrombus) AND (warfarin); (LV thrombus) AND ((anticoagulation) OR (vitamin K antagonist)); and (left ventricular thrombus) AND (direct oral anticoagulant).” Two authors (S.L. and T.D.) independently reviewed 12,176 citations by their titles, of which 6311 were duplicates and were excluded. The authors screened 3798 articles by titles, of which 3355 were excluded as they were not relevant to the outcome of interest, leaving 443 full-text articles to be assessed for eligibility. A total of 433 articles consisted of irrelevant patient populations (pediatric patients and young adults), case reports/ series, letters & editorials, reviews, and irrelevant study end-points (not pertaining to our pre-specified objectives), and thus were excluded. Ten studies were included in a qualitative synthesis, of which two studies were excluded because one study compared warfarin vs enoxaparin and not DOACs, while the other one was a duplicate. Finally, 8 articles met the criteria and were quantitatively evaluated (Fig. 1). Any conflicts pertaining to study selection were resolved by a mutual consensus [16]. All the published full-text articles and abstracts comparing warfarin with DOACs for the treatment of LVT were included in this systematic review, whereas studies that did not report outcomes stratified according to warfarin and DOACs cohort were excluded. We also excluded articles (case-reports and -series) comparing two treatments on a case-by-case basis.

### Data extraction and quality assessment

S.L. extracted the data from studies included in the quantitative analysis and recorded data on following variables of interest: author(s) name(s); region; year; duration; sample size; primary end-point; bleeding complications; stroke or systemic embolization; thrombus resolution; and mortality. T.D. did the review and quality appraisal. The quality appraisal was performed by using MINORS (Methodological Index for Non-Randomized Studies) scale that incorporates eight methodological parameters scored from 0 to 2 [17]. The overall score of included studies varied from 5 to 11. The results are shown in e-Table 2, Supplement–2.

### Data synthesis and analyses

Categorical variables between the two groups were summarized using the Mantel-Haenszel risk ratio (RR) and odds ratio (OR) along with their corresponding 95% CI and *p*-values [18, 19]. The pooled estimates were calculated by using a random-effects model for meta-analysis. For calculating tau-square (τ^2^), we used Hartung-Knapp-Sidik-Jonkman (HKSJ) method as it is known to perform better with fewer number of studies and has lower type-I error rates even when combining studies with unequal sample size [20]. Descriptive values reported as median [interquartile range] were converted to mean ± standard deviation by using Wan method [21]. Publication bias was evaluated by constructing funnel plots and Galbraith plots (as shown in eFigure in Supplement-2) and by performing Egger’s linear regression test of funnel-plot asymmetry. Between study heterogeneity was quantified with Higgins I^2^ statistic. We also constructed Baujat plots (see eFigure in Supplement-2) and performed an influential analysis to identify the presence of any outliers. A two-sided *p*-value < 0.05 was considered for defining statistical significance. We used *meta* and *metafor* packages for performing our meta-analyses [22, 23]. All statistical analyses were conducted in R (v3.6.3).

## Results

### Baseline demographic findings

We identified a total of 8 studies [12–14, 24–28] with a pooled sample size of 1955 patients comparing the therapeutic effectiveness and risk profile of warfarin vs DOACs in patients with LVT. The mean age of patients was 61 years and 59.7 years in warfarin and DOACs group, respectively. The proportion of males was similar between the two groups ranging from 57 to 62%. The prevalence of hypertension (16–75%) and smoking (21–60%) was similar between two groups, but there was higher variability in the prevalence of diabetes in DOACs group (8–86%) compared to warfarin group (16–40%). The studies included and baseline characteristics are shown in Table 1.

### Risk of bleeding complications and stroke or systemic embolization

**Fig. 2:**
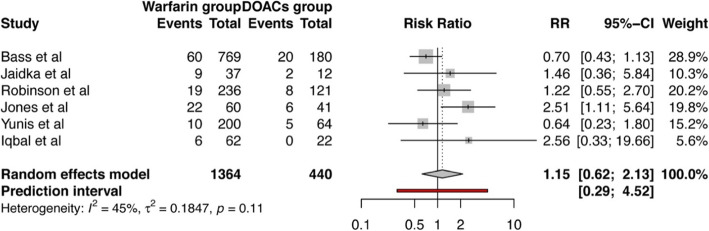
Forest plot showing pooled risk ratio of bleeding complications in patients treated with warfarin compared to those treated with DOACs for left ventricular thrombus

**Fig. 3:**
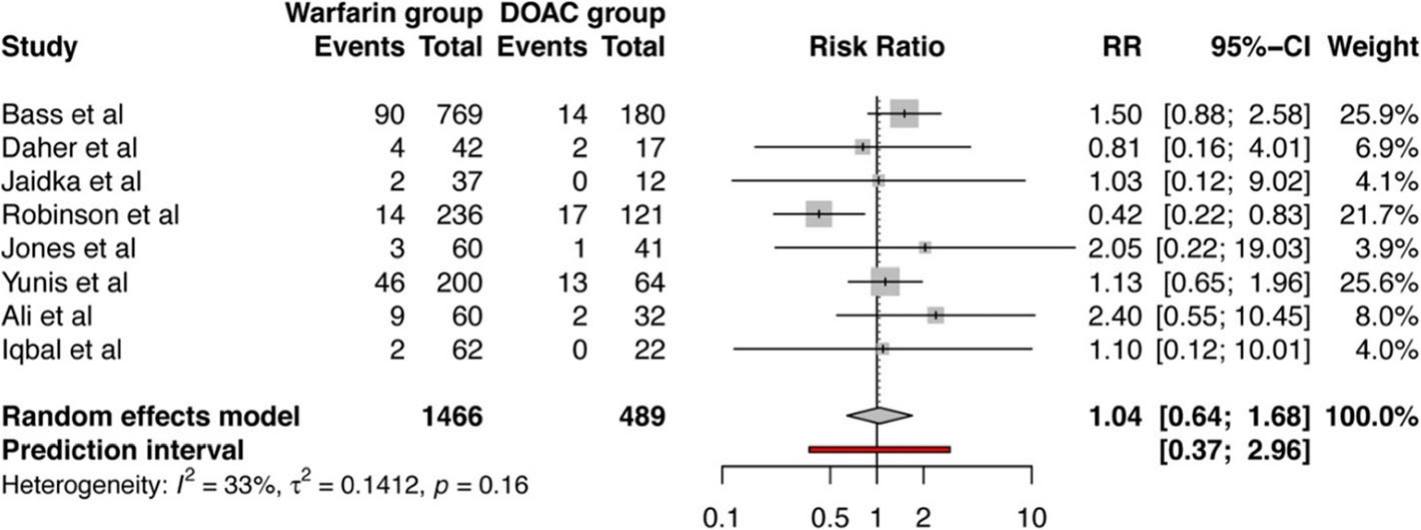
Forest plot showing pooled risk ratio of stroke or systemic embolization in patients treated with warfarin compared to those treated with DOACs for left ventricular thrombus

A total of 6 studies measured the occurrence of bleeding events in their analysis [12, 13, 24–27] using varying criteria to identify and record bleeding incidents: Bass et al. used The Global Use of Strategies To Open Occluded Coronary Arteries (GUSTO) criteria and blood product administration [29]; Jones et al. used Bleeding Academic Research Consortium (BARC) criteria [30]; Jaidka et al. classified bleeding as major and minor; while no criteria was specified by Robinson et al. and Yunis et al. We found that the pooled risk ratio (RR) of bleeding complications in patients treated with warfarin to those treated with DOACs was 1.15 (95% CI 0.62–2.13) (*p* = 0.57) as shown in Fig. 2. All included studies looked into occurrence of stroke or systemic embolization. The pooled RR for stroke or systemic embolization was — 1.04 (95% CI 0.64–1.68) (*p* = 0.85) for warfarin vs DOACs group (Fig. 3). No statistical significance was reached in either of above outcomes between two groups.

### Thrombus resolution

**Fig. 4:**
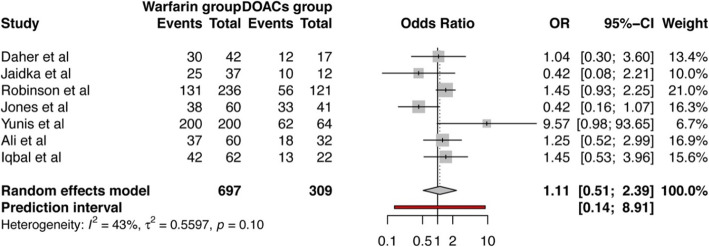
Forest plot showing pooled odds ratio of thrombus resolution in patients treated with warfarincompared to those treated with DOACs for left ventricular thrombus

Seven studies had reported their rates of thrombus resolution [12–14, 25–28]. Daher et al., Robinson et al., Jaidka et al., and Ali et al. relied on transthoracic echocardiogram (TTE) to measure LVT resolution; Jones et al. used TTE or cardiac magnetic resonance (CMR); and Iqbal et al. used CMR to diagnose LVT at the baseline with subsequent assessments made by TTE. The odds of thrombus resolution in warfarin group was 11% higher compared to DOACs group, with a pooled odds ratio of 1.11 (95% CI 0.51–2.39) but it did not reach a statistical significance (*p* = 0.76), as shown in Fig. 4.

### Mortality risk

**Fig. 5:**
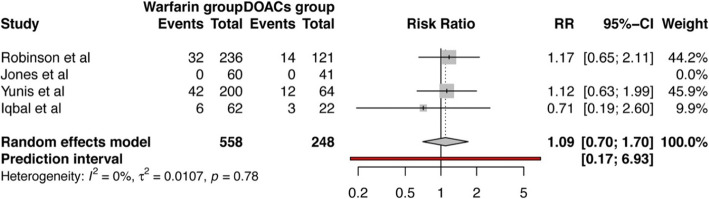
Forest plot showing pooled risk ratio of all-cause mortality in patients treated with warfarin compared to those treated with DOACs for left ventricular thrombus

We also calculated the pooled risk for overall mortality between the two groups. The risk of mortality was 9% higher in patients who received warfarin for LV thrombus compared to those who were treated with DOACs — RR 1.09 (95% CI 0.70–1.70), however the difference was not statistically significant with a *p*-value of 0.48 (Fig. 5).

## Discussion

To the best of our knowledge, this is the first metanalysis to compare safety and efficacy of DOAC vs warfarin in patients with LVT. There are multiple findings in our study that warrant emphasis. We found that there is no statistically significant difference in thrombus resolution, risks of bleeding complications, stroke or systemic embolization (SSE), and mortality in LVT patients treated with warfarin compared to those treated with DOACs. These findings suggest that DOAC is non-inferior or at least as effective as warfarin in the treatment of left ventricular thrombus. These results are consistent with results seen in some prior retrospective studies, where safety and efficacy of DOAC was comparable to warfarin for SSE prevention [12, 14, 24, 25].

Our results are contrary to those of Robinson et al., who reported that treatment with DOACs was associated with a higher risk of SSE compared to warfarin [13]. It is important to note that in this study, DOAC group had more patients with traditional risk factors of stroke like history of prior SSE, hyperlipidemia, atrial fibrillation, apical thrombus and pedunculated thrombus which could have contributed to more SSE events. Moreover, due to retrospective nature of this study, there can be some unmeasured confounders leading to increased SSE events in their study. We did not find such an association in our pooled analysis. On the other hand, Jones et al. found increased resolution of thrombus with DOACs [27]. Our pooled analysis did not show this association either.

Although LVT may occur in both ischemic and non-ischemic cardiomyopathies, their incidence is relatively high following an ST-segment elevation myocardial infarction (STEMI), particularly those involving the anterior wall [31, 32]. The pathogenesis involves endothelial dysfunction, akinesia occurring following myocardial tissue necrosis, and is composed of red blood cells, fibrin, and platelets [33]. LVTs are notorious because they carry a substantial risk of systemic embolization often resulting in a stroke. A previous meta-analysis (1993) elucidated that the odds ratio for the risk of mural thrombus embolism was 5.45 (95% CI 3.02–9.83) in patients with anterior myocardial infarction [34]. In a more recent study, Maniwa et al. (2018) observed that merely the presence of LVT was an independent predictor of systemic embolism (HR 4.00; 95% CI 2.11–7.23; *p* < 0.001) [7]. Not surprisingly, with adequate anticoagulation the occurrence of embolic events can be decreased [7, 35].

DOACs are a newer class of anticoagulants — with the first agent approved by the Food and Drugs Administration (FDA) in 2010. DOACs are indicated for the treatment of conditions such as non-valvular atrial fibrillation and venous thromboembolism. Factor Xa inhibitors, including apixaban, edoxaban and rivaroxaban, act by competitively inhibiting factor Xa in the common pathway of coagulation cascade, thereby preventing the formation of thrombin. Dabigatran, a direct thrombin inhibitor, reversibly inhibits both free and fibrin-bound thrombin resulting in inhibition of thrombin-mediated platelet aggregation [36]. In the recent years, off-label use of DOACs for the treatment of LVT has popularized among both physicians and patients owing to their ease of administration, lack of dietary restrictions, and freedom from regular blood draws [10, 11]. Moreover, multiple studies like RE-LY, ARISTOTLE, ROCKET-AF over the course of years have shown superiority or either non-inferiority of DOACs in patients with atrial fibrillation in preventing SSE with better bleeding profile [37–39]. Similarly, there is strong evidence supporting DOACs having similar or better efficacy than warfarin in deep venous thrombus resolution with less bleeding complications [40].

Hence due to aforementioned reasons, it is reasonable to think about using DOACs in LV thrombus patients. However, the safety profile and therapeutic efficacy of DOACs is debated with conflicting results, which is largely attributed to the lack of randomized controlled trials. Our study shows that the pooled odds ratio of thrombus resolution is 11% higher with 15% more risk of bleeding in warfarin group vs DOACs, however, neither outcome reached statistical significance. A recent systematic review of use of DOACs in the treatment of LVT included 53 articles and concluded that routine use of DOACs cannot be recommended based on their conflicting results [11]. However, it comprised largely of individual cases of LVT without quantitatively comparing the DOACs and warfarin.

The type (whether DOAC or vitamin K antagonist) and duration of anticoagulation treatment in patients with LVT is undetermined. The current guidelines laid by American College of Cardiology Foundation/American Heart Association (ACCF/AHA) for the management of STEMI gives a relatively weaker indication to anticoagulate LVT patients with vitamin-K antagonist (such as warfarin) for 3-months is setting of STEMI (Class IIa) [8]. Similarly, the European Society of Cardiology (ESC) recommends anticoagulation for 6-months along with repeated echocardiographic evaluation, bleeding risk assessment, and need for concomitant antiplatelet therapy (Class IIa) [9]. Due to limited data on safety and efficacy of DOACs they could not be included in guidelines. Study by Lattuca et al. suggests treating LV thrombus patients with > 3 months (regardless of type of anticoagulation) is associated with less major adverse cardiovascular events (MACE), which included death, stroke, myocardial infarction, or acute peripheral artery emboli [35].

Based on the findings of our analysis, although it may appear that the two treatments share a similar clinical and safety profile, we do not recommend using DOACs for left ventricular thrombus. The results of our study are hypothesis generating for further studies to evaluate the comparative effectiveness of DOACs compared to warfarin. The selection of anticoagulant in patient should be individualized based on risk-benefit discussion with the patient and the treating physician. Currently, there are few small prospective randomized trials comparing DOACs to warfarin in LVT patients under process and their results are eagerly awaited [41–44]. As they have small number of patients and limited follow up of 3 to 6 months, these studies may be underpowered to detect any significant differences. There is still a need of large randomized controlled trial for determining safety and efficacy of DOACs vs warfarin in patients with LV thrombus.

### Limitations

Our study has certain limitations that need to be addressed. First, largely the retrospective design of studies and lack of randomized trials included in the quantitative analysis. Second, the inclusion of individual studies with a relatively higher influence on the overall effect estimates and heterogeneity. The follow-up duration varied among various single-centered studies. Third, there was a paucity for data on the comparison of warfarin and DOACs, which could have been integral in our inability to reach a statistical significance. Fourth, due to limited information on duration to resolution of thrombus unable to conclude how long the patients should be treated. Readers of this meta-analysis should consider these limitations while interpreting the results and applying them to clinical practice.

## Conclusions

In summary, Vitamin-K antagonists such as warfarin are usually recommended for treating LVT, however our meta-analysis suggests treatment with DOACs also appears to be promising with equivalent efficacy and safety profile when compared to conventional treatment with warfarin. Hence, DOACs can be considered in patients with LV thrombus. However, large randomized studies comparing the effectiveness of DOACs to warfarin in LVT patients are needed to confirm these findings.

## Supplementary Information

**Additional file 1.**

## Data Availability

Not applicable.
